# County incidence and geospatial trends of early-onset hypertensive disorders of pregnancy in Kentucky, 2008-2017

**DOI:** 10.1186/s12884-023-05699-y

**Published:** 2023-06-19

**Authors:** Courtney J Walker, Anna M. Kucharska-Newton, Steven R. Browning, W. Jay Christian

**Affiliations:** 1 Department of Epidemiology, College of Public Health, University of Kentucky, Lexington, KY USA; 2 Department of Behavioral Sciences, University of Kentucky College of Medicine, CE Barnhart, Lexington, KY 40536 USA; 3 Department of Epidemiology, Gillings School of Global Public Health, University of North Carolina at Chapel Hill, Chapel Hill, NC USA

**Keywords:** Kentucky, Early-onset hypertensive disorders of pregnancy, Hypertensive disorders of pregnancy, Race, County trends, Geospatial analysis, Smoking during pregnancy

## Abstract

**Background:**

Early-onset hypertensive disorders of pregnancy (eHDP) are associated with more severe maternal and infant outcomes than later-onset disease. However, little has been done to evaluate population-level trends. Therefore, in this paper, we seek to address this understudied area by describing the geospatial and temporal patterns of county-level incidence of eHDP and assessing county-level demographics that may be associated with an increased incidence of eHDP.

**Methods:**

Employing Kentucky certificates of live and stillbirth from 2008–2017, this ecological study detected county-level clusters of early-onset hypertensive disorders of pregnancy using SaTScan, calculated average annual percent change (AAPC) with a join point analysis, and identified county-level covariates (% of births to women ≥ 35 years of age, % with BMI ≥ 30 kg/m^2^, % currently smoking, % married, and % experienced eHDP) with a fixed-effects negative binomial regression model for longitudinal data with an autoregressive (AR) correlation structure offset with the natural log of the number of births in each county and year.

**Results:**

County-level incidence of eHDP had a non-statistically significant increase of almost 3% (AAPC: 2.84, 95% CI: -4.26, 10.46), while maternal smoking decreased by almost 6% over the study period (AAPC:-5.8%, 95%CI: -7.5, -4.1), Risk factors for eHDP such as pre-pregnancy BMI ≥ 30 and proportion of births to women ≥ 35 years of age increased by 2.3% and 3.4% respectively (BMI AAPC:2.3, 95% CI: 0.94, 3.7; ≥ 35 years AAPC:3.4, 95% CI: 0.66, 6.3). After adjusting for race, county-level proportions of college attainment, and maternal smoking throughout pregnancy, counties with the highest proportion of births to women with BMI ≥ 30 kg/m^2^ reported an eHDP incidence 20% higher than counties with a lower proportion of births to mothers with a BMI ≥ 30 kg/m^2^ and a 20% increase in eHDP incidence (aRR = 1.20, 95% CI: 1.00, 1.44). We also observed that counties with the highest proportion vs. the lowest of mothers ≥ 35 years old (> 6.1%) had a 26% higher incidence of eHDP (RR = 1.26, 95%CI: 1.04, 1.50) compared to counties with the lowest incidence (< 2.5%). We further identified two county-level clusters of elevated eHDP rates. We also observed that counties with the highest vs. lowest proportion of mothers ≥ 34 years old (> 6.1% vs. < 2.5%) had a 26% increase in the incidence of eHDP (RR = 1.26, 95% CI: 1.04, 1.50). We further identified two county-level clusters of elevated incidence of eHDP.

**Conclusions:**

This study identified two county-level clusters of eHDP, county-level covariates associated with eHDP, and that while increasing, the average rate of increase for eHDP was not statistically significant. This study also identified the reduction in maternal smoking over the study period and the concerning increase in rates of elevated pre-pregnancy BMI among mothers. Further work to explore the population-level trends in this understudied pregnancy complication is needed to identify community factors that may contribute to disease and inform prevention strategies.

## Introduction

Hypertensive disorders of pregnancy (HDP) are a group of progressive diseases, occurring during pregnancy that includes gestational hypertension (GH), pre-eclampsia (PE), and eclampsia. From 2014-2017, HDP accounted for 6.6% of maternal deaths in the US and impacted an estimated 8-10% of US pregnancies [1, 2]. Maternal complications of HDP can include pulmonary edema, renal failure, stroke, and death [3–5]. Early-onset disease (eHDP), with symptom manifestation prior to 34 weeks gestation, have an increased risk of experiencing an HDP in future pregnancies and a higher risk and earlier onset of cardiovascular disease compared to those who experienced late-onset PE or normotensive pregnancies [6]. Treatments for any HDP are limited. For women perceived as "high risk," a daily low dose aspirin is recommended starting at 12 weeks gestation, but after the onset of symptoms, delivery is often the only option. However, premature delivery increases the infant's risk of poor health outcomes [7].

Although there are few trend assessments for eHDP, the overall incidence of HDP has been increasing. Between 1980 and 2003, HDP increased by an estimated 25% and states with a high incidence of BMI ≥ 30 kg/m^2 ^have reported some of the highest rates of HDP [8–10]. In a more recent one-year study assessing national trends, Kentucky, a state with elevated obesity and smoking rates, was identified as having the 8^th^ highest incidence of HDP. [8]. Regional differences observed in geospatial trends of HDP have been attributed to geographic variation in health behaviors and incidence of pre-existing conditions [11, 12]. However, limited research has been undertaken on the spatial temporal trends of HDP and eHDP incidence.

To address this gap, we used Kentucky birth records from 2008 to 2017 to explore spatiotemporal trends of eHDP, and identify county-level covariates associated with increased incidence of eHDP. We believe that areas with a high incidence of comorbidities such as elevated maternal BMI, and mothers of advanced age (> 35 years) will have an increased incidence of eHDP. Further, we will also compare the non-Appalachian and Appalachian region, as the central-Appalachian region has one of the highest burdens of chronic disease and poverty, factors that have been associated with an increased risk of pregnancy complications [13–16].

## Methods

### Data availability

This county-level ecologic study used electronic birth certificate data from vital records. The birth records data are not publicly available, per our data use agreement, but these data may be requested from the Commonwealth of Kentucky's Community for Health and Family Services branch [17]. Questions about data request parameters should be directed to Dr. Courtney Walker.

### Study population and outcome ascertainment

The Kentucky Department of Vital Statistics provided individual records for all live and stillbirths to self-identified Kentucky residents from January 1, 2008, through December 31, 2017 [17]. These records contained addresses, maternal information (date of birth, marital status, race, education, ethnicity, number of previous births, height, and pre-pregnancy weight), and pregnancy characteristics (gestation length, cigarettes smoked before and during each trimester of pregnancy, parity, number of previous pregnancies, and complications of pregnancy) and may be requested through the Kentucky Cabinet for Health and Family Services [17]. Although live and stillbirth forms differ slightly, all variables used in this study were recorded on both certificates [18]. We used individual records to identify singleton births to primiparous women (ages 12-50 years) between 20-45 weeks gestation, located in Kentucky. Records that did not geocode (*n* = 3) or indicate the mother had pre-existing chronic hypertension (*n* = 3,854) were excluded, as HDP and chronic hypertension are mutually exclusive on birth records [18, 19].

The birth form provides separate checkboxes for chronic hypertension, GH, and eclampsia [19]. Early-onset HDP (eHDP) was defined as check positive for GH on the birth certificate and birth between 20-34 weeks gestation. Rural**-**Urban Continuum Codes (RUCC) were obtained from the United States Department of Agriculture to characterize urban development at the county level [20]. Cartographic boundary files were obtained from the United States Census Bureau [21]. Appalachian status, defined by the Appalachian Regional Commission (ARC), was based on the geocoded maternal county of residence [22].

### Data cleaning and preparation

Maternal BMI was derived using the variables recorded on the birth record: mother's pre-pregnancy weight and height, and classified based on existing categories [23]. Smokers were defined as cigarette use throughout the entire pregnancy. Women who reported no smoking after the first trimester or no cigarette consumption were considered non-smokers, as the current literature suggests that women who stopped smoking during the first trimester had similar risks of HDP as those who were non-smokers [24, 25].

Each record was geocoded using the ESRI address coder (ESRI, Redlands, CA). Records with coordinates corresponding to the "rooftop" or a street segment were classified as "precisely geocoded." Imprecise coordinates were defind as those corresponding to the midpoint of a street, ZIP code, or city [26]. Using standard geocoding convention, we considered counties with more than 85% of addresses geocoded precisely as high precision; otherwise, they were classified as less precise areas [26]. For further details, see Walker et al. [27].

To better understand population-level trends we created a county-level dataset, using the individual-level geocoded records, to characterize the yearly county-level incidence of mothers of advanced maternal age (% ≥ 35 years old), race (% Black), ethnicity (% Hispanic), educational attainment (% completed college), marital status (% married), pre-existing diabetes (%), maternal BMI ≥ 30 kg/m^2^ (%), maternal smoking throughout pregnancy (%), and stillbirths (%). Incidence estimates were then classified into quartiles using PROC RANK, which creates categories based on the distribution of the variable and pre-specified number of categories [28]. Rurality status, geocoding precision, and Appalachian designation was determined at the county level [13, 20].

### Statistical analysis

#### Summary statistics

We summarized all covariates of interest as counts and percentages. To calculate the weighted average eHDP cases within each covariate level, we used the LS MEANS option within the PROC GENMOD with a negative binomial distribution and a log link.

#### Bivariate and multivariable models

To screen for multicollinearity, we calculated Spearman's rank pairwise correlation. No two variables had a rho greater than 0.6.

For both the bivariate and multivariate models, we fit a fixed-effects negative binomial regression model for longitudinal data with an autoregressive (AR) correlation structure, offset with the natural log of the number of births in each county and year using PROC GENMOD. Time was treated as a categorical variable in all models. Fixed effects allowed us to adjust for repeat measures (i.e. county trends). The negative binomial model was selected because the mean and variance structure assumption was violated for the Poisson model. The AR correlation structure was chosen because it allows for a stronger correlation between temporally closer times, and the strength of association is assumed to reduce as distance among time points increases.

For the bivariate model, initially, we used the negative binomial described above. We assessed each covariate interacted with time (categorical) to explore eHDP incidence in relation to the changes in each covariate over time individually, however, none of the interactions were statistically significant; therefore, we report the results without interactions.

For the final model, variables identified in the literature as important individual covariates, as there has been limited population-level studies of eHDP, were included in the base model [|maternal age ≥ 35 years (%), race (Black %), maternal BMI ≥ 30 kg/m^2^ (%), and smoking throughout pregnancy(%)]. All other covariates were removed with backward elimination. Variables that were statistically significant or changed the estimates of statistically significant covariates by more than 15% were retained in the model. The final model included maternal age ≥ 35 years (%), race (Black %), educational attainment (% completed college), marriage(%), maternal BMI ≥ 30 kg/m^2^ (%), smoking throughout pregnancy(%), and Appalachian region.

All analyses were conducted using used SAS v 9.4 (SAS Corp., Cary, NC). P-values less than 0.05 were considered statistically significant.

#### Mapping and temporal trend assessment

To explore geographic patterns of eHDP and detect and evaluate the statistical significance of any identified clusters, we performed unadjusted retrospective space**-**time cluster analyses using SaTScan (v 9.5) software. SaTScan™ is a trademark of Martin Kulldorff. The SaTScan™ software was developed under the joint auspices of (i) Martin Kulldorff, (ii) the National Cancer Institute, and (iii) Farzad Mostashari of the New York City Department of Health and Mental Hygiene. Briefly, this method delineates several overlapping cylinders of varied sizes and widths over the study area to identify possible clusters of cases in space and time [29]. For this study, each cylinder was centered on a point in a regular 5-mile grid and could encompass various surrounding counties. Generally, each cylinder's radius corresponds to geographic distance, and the height corresponds to time. Our study focused only on high-incidence clusters that contained at least two neighboring counties with eHDP. Maximum spatial cluster size was initially set to 30% of the study area population, as this would capture large cities, such as Louisville. However, no clusters were identified in urban areas, and the identified clusters were too large to be useful (e.g., 42 counties). Therefore, the maximum size of the spatial clusters was gradually reduced by five percent until the number of counties identified was narrow enough to identify potential areas for intervention. The final spatial cluster size was 10% of the covariate-adjusted population at risk. We also assessed purely spatial clusters to identify counties that may have an overall elevated rate of eHDP. Under the null hypothesis, we assumed that cases were Poisson distributed and risk was constant over space and time. The alternative hypothesis was that the risk would be higher inside the cluster than outside the cluster.

We created choropleth maps using QGIS (Madeira v 3.4) to display identified clusters and visualize the average incidence of eHDP and incidence of maternal BMI ≥ 30 kg/m^2^, maternal smoking throughout pregnancy, and marital status at birth for each county throughout the study. We used the Jenks method to determine categories for choropleth maps [30].

We used a general linear estimation (GLM) model with a Poisson distribution and a log link to obtain yearly estimates of eHDP and the average annual percent change (AAPC). Significant covariates (maternal BMI ≥ 30 kg/m^2^, smoking throughout pregnancy, marriage, and eHDP) were assessed for significant inflection points using Joinpoint software.

The Medical Institutional Review Board at the University of Kentucky and the Kentucky Cabinet for Health and Family Services (CHFS) Institutional Review Board approved this protocol (Protocol 44968, Approved 10/26/2018). As this study accessed data routinely collected in birth certificates, the IRB waived the requirement for informed consent. While they did not contain names, medical record numbers, or social security numbers, these data were not fully anonymous as they included full addresses for all births. Strengthening the Reporting of Observational Studies in Epidemiology (STROBE) guidelines were used as a reporting template [31].

## Results

### Summary statistics

In this retrospective ecological study, we observed 1,936 cases of eHDP among 212,544 births (9.1 cases per 1,000 births) in Kentucky from 2008–2017. Table 1 displays the marginal means of eHDP for each subgroup. Counties with the highest incidence of Black mothers had the lowest average number of eHDP cases (8.1, 95%CI: 7.4, 9.0), as did the areas with the lowest BMI ≥ 30 kg/m^2^ incidence (7.9, 95%CI: 7.2, 8.5). The Appalachian region had one of the highest marginal means, with 11.1 eHDP cases per 1,000 births (95%CI: 10.2, 12.0).

**Table 1:** Average eHDP incidence in Kentucky by demographic group, 2008-2017

	County level average incidence of eHDP (95%CI)
Maternal age ≥ 35 years old (%)	
< 2.5	10.75 (9.45, 12.00)
2.5 to 4.2	10.15 (9.20, 11.50)
4.2 to 6.1	9.95 (9.00, 11.00)
> 6.1	8.35 (7.70, 9.00)
Race (Black %)	
0	10.45 (9.30, 11.50)
0.1-1.7	10.05 (8.95, 11.50)
1.7 to 3.4	9.80 (8.70, 11.00)
3.4 to 6.2	10.05 (9.00, 11.50)
> 6.2	8.10 (7.40, 9.00)
Educational attainment (% completed college)	
< 18.0	9.95 (8.70, 11.50)
18.0 to 23.4	10.55 (9.45, 12.00)
23.4 to 30.2	9.60 (8.60, 10.50)
> 30.2	8.75 (8.10, 9.50)
Marriage (%)	
< 43.6	8.90 (7.70, 10.50)
43.6 to 48.6	9.70 (8.75, 11.00)
48.6 to 53.8	9.35 (8.50, 10.50)
> 53.8	9.75 (8.90, 10.50)
Maternal BMI ≥ 30 kg/m^2^ (%)	
< 22.6	7.95 (7.25, 8.50)
22.6 to 26.8	9.70 (8.80, 10.50)
26.8 to 31.6	10.15 (9.15, 11.50)
> 31.6	11.20 (10.00, 12.50)
Maternal smoking throughout pregnancy (%)	
< 13.2	8.45 (7.80, 9.00)
13.2 to 17.8	9.80 (8.85, 11.00)
17.8 to 23.0	10.95 (9.85, 12.00)
> 23.0	9.90 (8.70, 11.00)
Appalachian Region	
Non-Appalachian	8.70 (8.15, 9.50)
Appalachian	11.10 (10.20, 12.00)
Year	
2008	8.10 (6.85, 9.50)
2009	8.45 (7.20, 10.00)
2010	9.10 (7.75, 10.50)
2011	8.50 (7.20, 10.00)
2012	9.45 (8.05, 11.00)
2013	10.90 (9.40, 12.50)
2014	9.60 (8.20, 11.50)
2015	10.30 (8.75, 12.00)
2016	10.10 (8.60, 12.00)
2017	10.45 (8.90, 12.50)

### Bivariate and multivariate models

Table 2 displays the unadjusted and adjusted models. In the unadjusted model, we observed that counties with the lowest proportion of mothers ≥ 35 years old had a 27% higher incidence of eHDP (RR = 1.27, 95% CI: 1.08, 1.52) than counties with the highest proportion of mothers ≥ 34 years old. Also of note in the unadjusted model was the 33% reduction in eHDP incidence in counties with the highest percentage of Black mothers compared to counties with no Black mothers (RR = 0.77, 95% CI: 0.64, 0.94).

**Table 2:** Unadjusted and adjusted incidence of eHDP by demographic category

	**RR (95%CI)**	***p*-value**	**aRR** **(95%CI)**	***p*-value**
Mother ≥ 35 years old (%)				
< 2.5	1.20 (1.00, 1.45)	0.04	1.10 (0.94, 1.32)	0.24
2.5 to 4.2	1.23 (1.04, 1.46)	0.03	1.14 (0.98, 1.30)	0.084
4.2 to 6.1	1.11 (0.94, 1.31)	0.03	1.00 (0.86, 1.14)	0.978
> 6.1	Reference		Reference	
Race (Black %)				
> 6.1	0.77 (0.64, 0.94)	0.01	1.04 (0.86, 1.26)	0.657
3.4 to 6.1	0.96 (0.80, 1.15)	0.69	1.18 (1.00, 1.38)	0.054
1.7 to 3.4	0.94 (0.77, 1.14)	0.55	1.10 (0.94, 1.3)	0.249
0.1–1.7	0.90 (0.81, 1.14)	0.69	1.02 (0.88, 1.18)	0.825
0	Reference		Reference	
Educational attainment (% completed college)				
> 30.2	0.80 (0.73, 1.05)	0.18	1.00 (0.82, 1.22)	0.969
23.4 to 30.2	0.95 (0.80, 1.14)	0.63	0.92 (0.78, 1.10)	0.381
18.0 to 23.4	1.00 (0.91, 1.22)	0.43	1.00 (0.86, 1.18)	0.954
< 18.0	Reference		Reference	
Marriage (%)				
> 53.8	1.10 (0.93, 1.30)	0.24	1.36 (1.14, 1.60)	0
48.6 to 53.8	1.05 (0.89, 1.25)	0.51	1.16 (1.00, 1.36)	0.063
43.6 to 48.6	1.10 (0.90, 1.34)	0.34	1.18 (1.02, 1.38)	0.033
< 43.6	Reference		Reference	
Maternal BMI ≥ 30 kg/m^2^ (%)				
> 30.3	1.40 (1.17, 1.69)	< 0.01	1.26 (1.04, 1.50)	0.012
25.7 to 30.3	1.27 (1.08, 1.40)	< 0.01	1.12 (0.96, 1.30)	0.171
21.5 to 25.6	1.21 (1.04, 1.42)	0.01	1.12 (0.98, 1.30)	0.105
< 21.5	Reference		Reference	
Maternal smoking throughout pregnancy (%)				
> 23.2	1.16 (0.97, 1.39)	0.09	1.16 (0.96, 1.42)	0.12
17.9 to 23.2	1.29 (1.10, 1.51)	< 0.01	1.30 (1.10, 1.54)	0.003
13.2 to 17.8	1.10 (0.96, 1.38)	0.12	1.16 (1.00, 1.32)	5
< 13.2	Reference		Reference	
Appalachian Region				
Non-Appalachian	0.78 (0.68, 0.89)	< 0.01	0.86 (0.76, 0.98)	0.02
Appalachian	Reference		Reference	
Year				
2017	1.30 (1.07, 1.57)	0.01	1.36 (1.10, 1.68)	3
2016	1.25 (1.05, 1.49)	0.01	1.40 (1.16, 1.68)	0
2015	1.27 (1.05, 1.54)	0.01	1.36 (1.14, 1.62)	0
2014	1.19 (0.97, 1.45)	0.09	1.30 (1.06, 1.60)	0.012
2013	1.35 (1.10, 1.65)	< 0.01	1.48 (1.24, 1.78)	0
2012	1.17 (0.93, 1.45)	0.17	1.22 (1.00, 1.50)	0.045
2011	1.05 (0.84, 1.31)	0.64	1.10 (0.90, 1.34)	0.348
2010	1.13 (0.93, 1.37)	0.22	1.20 (1.00, 1.44)	0.042
2009	1.04 (0.85, 1.27)	0.66	1.12 (0.92, 1.36)	0.243
2008	Reference		Reference	

In the final model, adjusted for mothers ≥ 35 years old (%), race (Black %), marriage (%), maternal BMI ≥ 30 kg/m^2^ (%), maternal smoking throughout pregnancy (%), the Appalachian region, and year, we observed that low proportions of mothers ≥ 35 years old, and higher proportions of maternal BMI ≥ 30 kg/m^2^ (%) and marriage (%) were associated with an increased incidence of eHDP. In the unadjusted model, race showed a statistically significant decrease in eHDP incidence; however, in the adjusted model, this relationship shifted to a non-significant *increase* in incidence compared to counties with no Black mothers (RR = 1.04, 95% CI: 0.86, 1.26). The proportion of married mothers, insignificant in the unadjusted model, increased the incidence of eHDP by 36% in the adjusted model (RR = 1.36, 95% CI: 1.16, 1.60). The relative risk of eHDP in the non-Appalachian region, compared to the Appalachian region, remained similar following covariate adjustment (RR: 0.78, 95% CI: 0.68, 0.89, aRR: 0.86, 95% CI: 0.76, 0.98).

### Mapping and temporal trend assessment

**Fig. 1:**
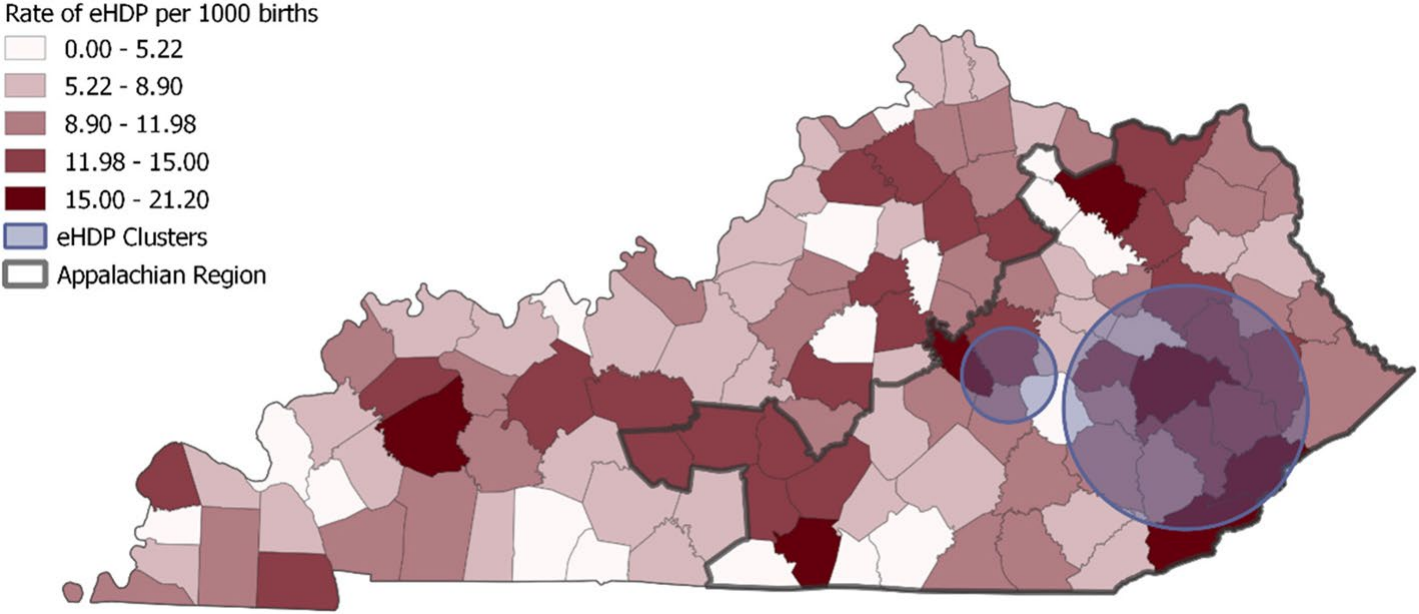
Choropleth map of rates of average eHDP rates over the study period and high rate clusters

Two clusters were identified in the spatial analyses (Fig. 1). The largest cluster was comprised of 14 counties (Table 3). Three counties in the largest cluster had more than 15 eHDP cases per 1,000 births throughout the study period (2008–2017). The smallest cluster of eHDP, comprised of two counties, was limited to only one year, 2012.

**Table 3:** Details of identified clusters of elevated eHDP incidence, 2008-2017

**Cluster description Counties**	**Years**	**In cluster case | pop**	Out cluster **case | pop**	**RR**	***P*-value**
**1. Large cluster – Eastern KY:**	2008**-**2017	148 | 1083	1784 | 18,339	1.54	0.03
Breathitt, Perry, Knott, Magoffin, Leslie, Owsley, Wolfe, Lee, Clay, Floyd, Morgan, Letcher, and Harlan					
**2. Small cluster – Central KY:** Madison and Rockastle	2012	18 | 466	176 | 20,830	3.81	0.05

*N* Number, *RR* Relative Risk, *Pop* Population

**Fig. 2:**
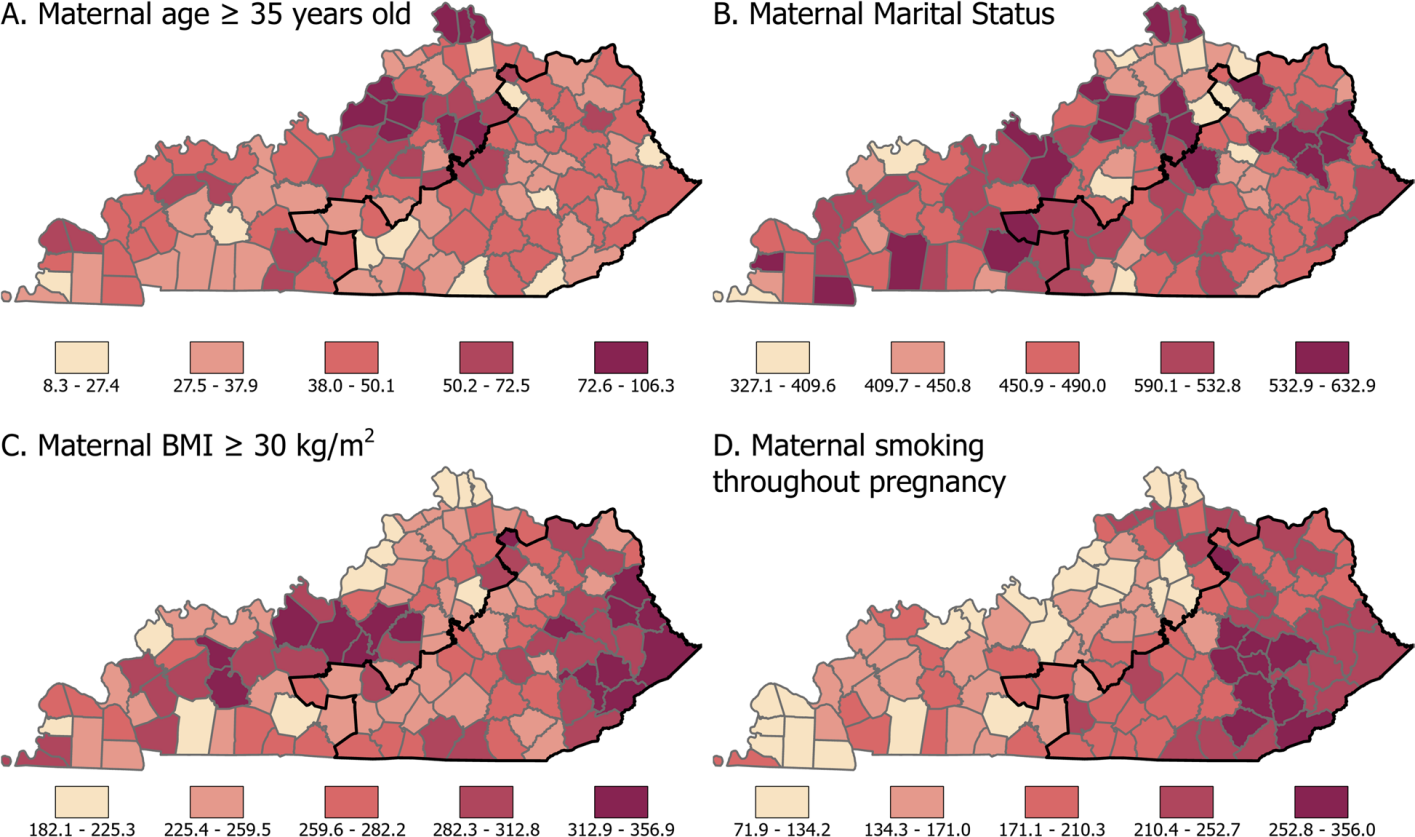
Choropleth maps of county-level proportions of, births to women ≥ 35 years of age (**A**) marriage (**B**) maternal BMI ≥ 30 kg/m^2 ^(**C**), and maternal smoking (**D**) per 1000 births in Kentucky, 2008–2017

Choropleth maps were created to visualize the incidence of maternal BMI ≥ 30 kg/m^2^, marriage, maternal age ≥ 35, and current maternal smoking per 1000 births (Fig. 2). We assessed the residuals for each county and year with Moran's I with GeoDa (v 1.18, December 2020). There was no indication of patterns of poor model fit [32].

There was a non-statistically significant increase in the incidence of eHDP (AAPC: 2.8, 95% CI: -4.3, 10.5) over the study period (Table 4). Both incidence of maternal BMI ≥ 30 kg/m^2^ (AAPC: 2.2, 95% CI: 0.8, 3.6) and births to mothers ≥ 34 years (AAPC: 2.9, 95% CI: -0.3, 6.2) increased over the study period. Maternal smoking decreased by almost 6% (AAPC: -5.9%, 95% CI: -7.6, -4.2). Upon visual inspection, there appeared to be a shift in the incidence of BMI ≥ 30 kg/m^2^ and smoking in 2012; however, further investigation using Joinpoint (Version 4.8.0.1) to assess inflection points yielded non-significant results.

**Table 4:** Incidence of eHDP, marriage, maternal BMI ≥ 30 kg/m^2^, and maternal smoking per 1000 births in Kentucky, 2008-2017

**Year**	**eHDP**	**Married**	BMI** ≥ 30 kg/m**^**2**^	**Smoking during pregnancy**	Maternal age** ≥ 35** years
2008	7.70	504.18	223.26	185.90	43.76
2009	8.22	501.52	223.86	166.94	45.42
2010	8.70	503.92	221.08	154.48	41.96
2011	8.12	499.32	230.80	151.24	45.02
2012	9.02	505.18	233.42	155.26	45.14
2013	10.72	504.74	233.38	143.10	45.80
2014	9.50	513.70	243.38	128.12	47.62
2015	9.80	513.58	252.90	119.58	52.76
2016	9.78	514.00	257.04	111.30	53.22
2017	9.90	521.36	270.44	99.70	55.68
AAPC (95%CI)	2.84 (-4.26, 10.46)	0.40 (-0.56, 1.36)	2.20 (0.78, 3.62)‡	-5.94 (-7.64, -4.20)‡	2.90 (-0.28, 6.18)†

*eHDP* Early-onset HDP (HDP onset < 34 weeks), *AAPC* Annual Average percent change, *CI* Confidence Interval, † *p*-value < 0.05, ‡ *p*-value < 0.01

## Discussion

Early-onset hypertensive disorders of pregnancy (eHDP) is a severe progression within hypertensive disorders of pregnancy (HDP) and is associated with short-term increased risk of maternal complications, and often has no treatment options except pre-term delivery, which increases the risk of poor infant outcomes [6, 7]. 

This retrospective ecological study sought to characterize the incidence of eHDP in a state with a high prevalence of potential risk factors, identify significant county-level covariates associated with increased eHDP incidence, and describe geospatial patterns of eHDP in Kentucky. This study observed that a low county-level incidence of primiparous mothers ≥ 35 years old and high county-level incidence of maternal BMI ≥ 30 kg/m^2 ^and smoking during pregnancy were associated with increased incidence of eHDP. Additionally, we detected two clusters of eHDP—one in the Appalachian region and one approaching statistical significance in Western Kentucky. This study confirms that the incidence of eHDP and pre-pregnancy BMI ≥ 30 kg/m^2^are increasing in Kentucky and has shown clear spatial patterns in eHDP incidence. These findings serve as an opportunity to identify areas that may need additional education to identify eHDP earlier, inform support needs for maternal support, and generate hypotheses that merit further study.

The incidence of eHDP in Kentucky from 2008 to 2017 was approximately 9.2 cases per 1,000 births. Although there are no nationwide estimates of eHDP incidence, a study using birth records from Washington reported 3.8 eHDP per 1,000 births – which are less than half of Kentucky rates [33]. These findings may reflect the general elevation of risk factors in Kentucky relative to Washington; however, further study is needed, as there are other notable differences between these states [34, 35]. We also found a 3% increase in eHDP incidence. In a study of regional trends, Wallis and colleagues reported that PE, a subset of HDP, increased by 29.4% and GH by 30.6% over 17 years (1987**-**2004). However, it is unclear if these increases reflect changes within the population or the multiple modifications of the case definition over the study period (1996, 2002, and 2013) [2, 9].

Unanticipated findings of this study were the increased incidence of eHDP found in communities with a high incidence of maternal smoking, as studies with individuals have reported that smoking throughout pregnancy has been found to decrease risk. Our findings may result from an ecological fallacy or could be a reflection of the predominance of other risk factors, such as a higher proportion of young mothers or higher of incidence of elevated BMI. These findings may reflect poor community health and low reproductive health literacy among young adults, especially in high-poverty regions. Further research exploring the impact of current health status and the risk of eHDP is needed.

This study also characterized overall maternal health in primiparous mothers. Specifically, we found maternal smoking incidence decreased by almost 6%, and pre-pregnancy BMI increased by over 2% over the study period. We also observed the Appalachian region, which includes 54 Kentucky counties, had eighteen counties where the incidence of maternal BMI ≥ 30 kg/m^2^was 30% or greater, twelve counties where the maternal smoking incidence was greater than 25%, and six counties with an eHDP incidence greater than 15 cases per 1000 births. In this study, we did not assess the causal associations between maternal BMI ≥ 30 kg/m^2^, smoking, and eHDP; however, further assessment of these relationships is needed.

### Strengths and limitations

There are some notable strengths of this study. The certificates of live and stillbirth are established administrative data collection forms that underwent routine quality control and remained relatively unchanged throughout the study period. Important demographic information (maternal age, race, ethnicity, and education) and pregnancy characteristics (gestation, prenatal care, pre-pregnancy weight, height, and gestational age at birth) are routinely collected [36–38]. We had sufficient study power to detect a statistically significant spatio-temporal cluster of eHDP, an infrequent pregnancy complication.

However, this study had noted limitations. Maternal demographics, pregnancy characteristics, and birth outcomes information came from birth records, which could be impacted by interviewer and recall bias. Birth records have been shown to have underreporting biases with other pregnancy conditions [39]. Further, our case definition was based on HDP, as we could not distinguish among subsets of HDP and gestation at disease onset, as the latter was not reported on the form [19]. This may result in some misclassification, as some women may have eHDP but delivered > 34 weeks. Birth records collection has also been shown to vary among Kentucky hospitals [40]. We believe any biases introduced due to clerical error are non-differential as there is no indication that misclassification occurred based on any discernible patterns [40, 41].

Geocoding maternal addresses is another source of potential bias in this study, as the previous research has shown that Kentucky birth records in rural areas geocode less precisely than their urban counterparts [27]. However, given the overall precision of the records and the spatial unit of analysis at the county level, we believe that the impact of geocoding imprecision on cluster identification is limited. However, residency changes, which are not reported on the birth certificate, may have led to non-differential misclassification of women who changed residence during pregnancy [42].

## Conclusion

In this ecological study, we have detected two county-level clusters of eHDP, identified county-level covariates associated with increased incidence of eHDP, and assessed trends of eHDP and covariates associated with eHDP. While increasing, the average rate of increase for eHDP was not statistically significant, however, did detect a decrease in maternal smoking and the concerning increase in high pre-pregnancy BMI among mothers. Further work is needed to identify causal factors associated with disease and inform prevention strategies.

## Data Availability

The birth records data are not publicly available but may be requested from the Commonwealth of Kentucky's Community for Health and Family Services branch [18].
